# A Novel Highly Sensitive NO_2_ Sensor Based on Perovskite Na_0.5+x_Bi_0.5_TiO_3−δ_ Electrolyte

**DOI:** 10.1038/s41598-017-05169-4

**Published:** 2017-07-10

**Authors:** Yihong Xiao, Chufan Zhang, Xu Zhang, Guohui Cai, Yong Zheng, Ying Zheng, Fulan Zhong, Lilong Jiang

**Affiliations:** 1 National Engineering Research Center of Chemical Fertilizer Catalyst (NERC-CFC), School of Chemical Engineering, Fuzhou University, Gongye Road No. 523, Fuzhou 350002, Fujian, P. R. China; 2 College of Chemistry and Materials Science, Fujian Normal University, Fuzhou, China

## Abstract

NO_x_ is one of dangerous air pollutants, and the demands for reliable sensors to detect NO_x_ are extremely urgent recently. Conventional fluorite-phase YSZ used for NO_x_ sensor requires higher operating temperature to obtain desirable oxygen ion conductivity. In this work, perovskite-phase Na_0.5_Bi_0.5_TiO_3_ (NBT) oxygen conductor was chosen as the solid electrolyte to fabricate a novel highly sensitive NO_2_ sensor with CuO as the sensing electrode and Pt as reference electrode. Na dopped Na_0.5_Bi_0.5_TiO_3_ greatly improved the sensing performance of this sensor. The optimal sensor based on Na_0.51_Bi_0.50_TiO_3−δ_ exhibited good response-recovery characteristics to NO_2_ and the response current values were almost linear to NO_2_ concentrations in the range of 50–500 ppm at 400–600 °C. The response current value towards NO_2_ reached maximum 11.23 μA at 575 °C and the value on NO_2_ is much higher than other gases (CH_4_, C_2_H_4_, C_3_H_6_, C_3_H_8_, CO), indicating good selectivity for detecting NO_2_. The response signals of the sensor were slightly affected by coexistent O_2_ varying from 2 to 21 vol% at 575 °C. The response current value decreased only 4.9% over 2 months, exhibiting the potential application in motor vehicles.

## Introduction

As one of the most dangerous air pollutants NO_x_ has attracted great attention of environmentalists and researchers during the past decades due to its severe effect on human health and environment^1^. Policies and regulations to reduce NO_x_ emissions are more and more strict, especially those rules aiming at exhaust emission restriction of transport vehicles, which are major NO_x_ emission sources. Therefore, a reliable device which is capable of monitoring NO_x_ content in vehicle exhaust has been in an urgent demand recently.

Electrochemical sensor is one of reliable appliance which has drawn considerable attention for its advantage to detect NO_x_ accurately in high temperature and harsh operation of exhaust gases^1, 2^. As well known, amperometric, potentiometric and impedimetric type are the main mode of electrochemical sensors. The amperometric-type sensor can realize fast detection to NO_x_ concentration through monitoring in-suit current signal^3, 4^. This sensor mainly consists of solid electrolyte, sensing electrode and reference electrode, whereas the matching of solid electrolyte and sensing electrode would directly impact the sensing performance of NO_2_ sensor. Commercialized NO_2_ sensor based on YSZ solid electrolyte with NiO sensing electrode exhibits high sensing performance. However, the sensitivity of the sensor for selective detection at low and middle temperature (450–700 °C) is a lingering issue.

Based on the principle of amperometric-type NO_2_ sensor, the following electrochemical reactions occurred at three phase boundary (TPB) of cathode (sensitive electrode, SE) and the anode (Pt reference electrode, RE) have been proposed^4, 5^:

1 $$(SE)Cathode:N{O}_{2}+2{e}^{-}\to NO+{O}^{2-}$$

2 $$NO+2{e}^{-}\to \frac{1}{2}{N}_{2}+{O}^{2-}$$

3 $$(RE)Anode:{O}^{2-}-2{e}^{-}\to \frac{1}{2}{O}_{2}$$

The absorbed NO_2_ molecules on the surface of SE gain electrons and then generate nitric oxide and oxygen ion with potential between the SE and the RE fixed at a certain value. The generated NO would be further decomposed into N_2_ by gaining electrons due to the high catalytic activity of SE, which in turn promotes the generation rate of O^2−^ on SE. The O^2−^ generated at the SE is conducted from the cathode side to the Pt anode side through oxygen ion conductor. Then the O^2−^ loses electron and turns into O_2_. In whole process of the electrode catalytic reaction, the rate-determining step is closely related to the adsorption of the sensing electrode towards NO_2_ at TPB, the formation rate of O^2−^, and the transmission speed of O^2−^ generated at the cathode that is depended on the electrolyte material. Therefore, choosing an appropriate solid electrolyte that is extremely mated by SE is one of cored problems of NO_2_ sensor.

The performance of the SE would greatly affect the sensing speed and sensitivity of NO_2_ sensor by greatly promoting the adsorption of target gas and reduction of NO_2_ happened at TPB. Recently, p-type semiconducting metal-oxides have drawn a lot of attention as sensing electrode, such as NiO, TeO_2_, Co_3_O_4_ and CuO due to their easy acquisition and low cost^6–9^. Compared to other sensing electrode materials, the treatment temperature of CuO electrode is much lower, and it has been widely used as heterogeneous catalysis for the reduction of NO_x_^10, 11^. thus is more conducive to the adsorption and reduction of NO_2_.

Compared to YSZ, perovskite oxide (ABO_3_) can adapt to variation by different substituents both A site and B site, offering more possiblities of vacancy oxygen which can be greatly improved by doping modification^12, 13^. Several kinds of perovskite oxides have been used as electrolytes for NO_x_ sensor which perform higher sensing performance at low- and intermediate-temperature (~400–600 °C) than that of YSZ^14–17^, indicating the potential usage of perovskite oxide towards NO_x_ sensor. The perovskite oxide GdAlO_3_ used for NO_2_ sensor has been reported, whereas both response and recovery speed are still slow^17^. Among perovskite oxides, sodium bismuth titanate (Na_0.5_Bi_0.5_TiO_3_) is one of special ABO_3_ perovskite-phase oxide which has two atomics (Na and Bi) occupying A site. YS Sung^18, 19^, R Zuo^12^, M Naderer^13^ and Li^20, 21^ explored the influence of nonstoichiometry upon ion conductivity and doping modification to Na_0.5_Bi_0.5_TiO_3_ shows potential in oxide ion conduction. Li^21^ also reported that the Mg-doping Na_0.5_Bi_0.49_Ti_0.98_Mg_0.02_O_2.965_ exhibited excellent conductivity to ~0.01 S·cm^−1^ at 600 °C and predicted the possible application in SOFC and sensor field as electrolyte material. K Meyer and K Albe^22^ reported that the different phase of NBT has different migration barriers, and the association of oxygen vacancies with migration barriers can be used to explain the change in activation energy. Strangely, to our best knowledge, not an electrochemical NO_2_ sensor prepared by sodium bismuth titanate as electrolyte has been reported, while the greatly enhanced ion conductivity is a considerable superiority in the application of NO_2_ sensor.

**Figure 1:**
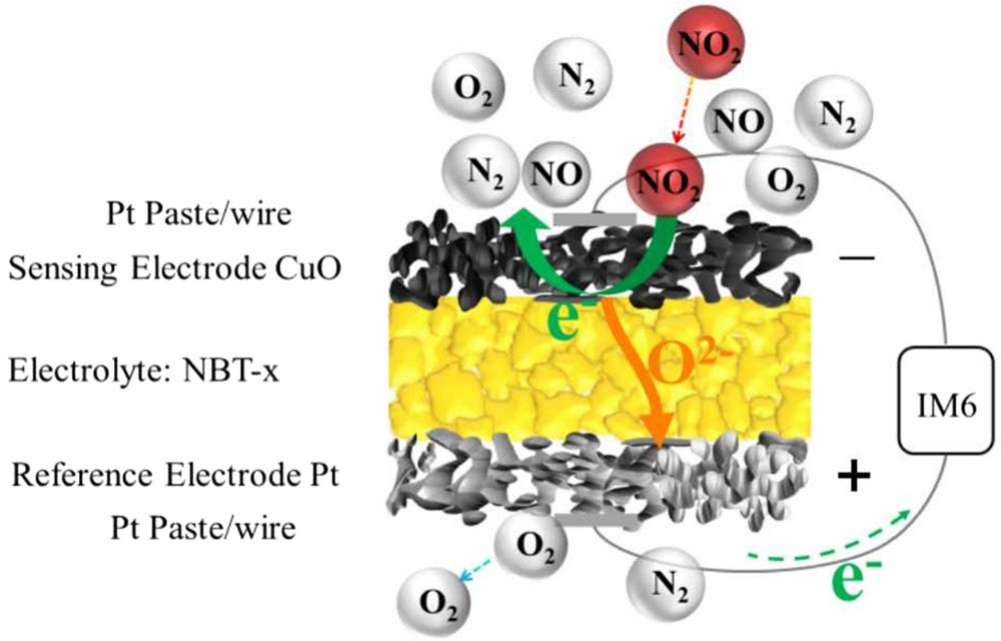
Schematic representation of the fabricated sensor.

In this work, a novel highly sensitive NO_2_ sensor based on perovskite-phase Na_0.5+x_Bi_0.5_TiO_3−δ_ (NBT, x = −0.01~0.04) electrolyte with CuO as the SE and Pt as RE was fabricated, as depicted in Fig. 1. The doping of nonstoichiometry Na greatly enhanced the sensing performance of NBT sensor. The optimal sensor based on NBT-0.01 exhibited good response-recovery characteristics to NO_2_ and the response current values towards NO_2_ reached maximum 11.23 μA at 575 °C, which was greatly higher than that of the sensor based on commercial YSZ with CuO SE (5.11 μA) under the same conditions, exhibiting its potential application with low cost in motor vehicles.

## Result and Discussion

**Figure 2:**
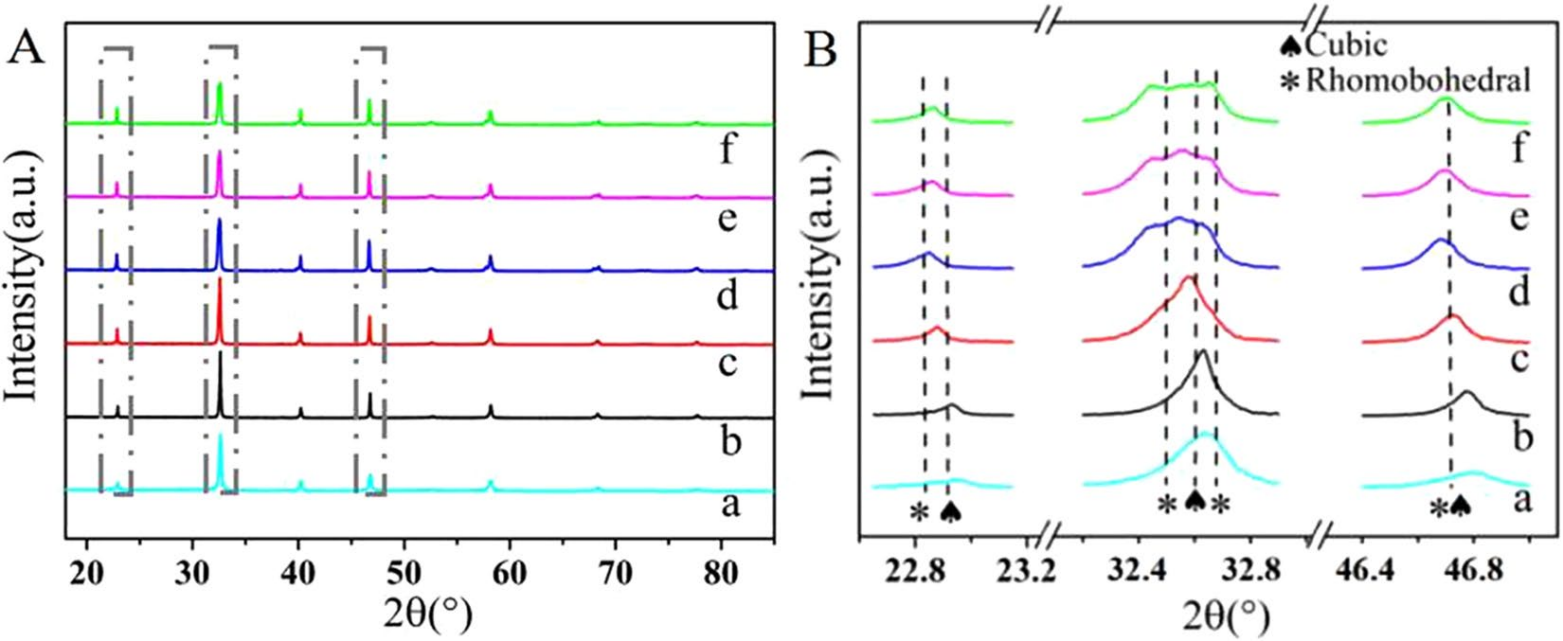
(**A**) X-ray diffraction patterns of Na_0.5+x_Bi_0.5_TiO_3−δ_ powers calcined at 1150 °C for 2 h; (**B**) Enlarged portion at 2θ = 22.6–23.2°, 32.3–32.9°, and 46.4–46.9°: (a) x = −0.01, (b) x = 0, (c) x = 0.01, (d) x = 0.02, (e) x = 0.03, (f) x = 0.04.

The Na_0.49_Bi_0.5_TiO_3−δ_, Na_0.50_Bi_0.5_TiO_3−δ_, Na_0.51_Bi_0.5_TiO_3−δ_, Na_0.52_Bi_0.5_TiO_3−δ_, Na_0.53_Bi_0.5_TiO_3−δ_, Na_0.54_Bi_0.5_TiO_3−δ_ of Na_0.5+x_Bi_0.5_TiO_3−δ_ (x = −0.01, 0, 0.01, 0.02, 0.03, 0.04) are denoted as NBT-(−0.01), NBT-0, NBT-0.01, NBT-0.02, NBT-0.03, NBT-0.04, respectively. Figure 2A shows the XRD patterns of Na_0.5+x_Bi_0.5_TiO_3−δ_ powders with different Na content calcined at 1150 °C. The main diffraction peaks of all compositions are identical with the standard XRD spectrum of Na_0.5_Bi_0.5_TiO_3_ (JCPDS 01–089–3109) or Na_0.5_Bi_0.5_TiO_3_ (JCPDS 00–036–0153), presenting a pure perovskite structure with a cubic or rhombohedral phase at room temperature, respectively. The sample of mentioned NBT−0.01 composition is a watershed. Basically the XRD spectrum of NBT-(−0.01) and NBT-0 belong to cubic phase, while the XRD patterns of NBT-0.02, NBT-0.03 and NBT-0.04 samples correspond to rhombohedral phase. The changes in stoichiometry at Na-site haven’t altered the basic perovskite structure, because no impurity phases except NBT can be found. The shifts of 2θ in the ranges of 22.6–23.2°, 32.3–32.9°, and 46.4–46.9° toward lower angle in Fig. 2B imply the lattice distortion, which probably induces variation in oxygen vacancies. The lattice constant values calculated by MDI Jade software were summarized in Table 1. As seen from Table 1, the lattice constant values are different from that of the original Na_0.5_Bi_0.5_TiO_3_, indicating that the variations of Na content bring changes to lattice structure. Since Na possesses similar ionic radius to Bi other than Ti, the changes caused by excess or deficient Na tend to occur in A-site^23^. The excessive Na can substitute a fraction of Bi, which will create larger A-site volume, because the ionic radius of Na is larger than that of Bi^24^. The overflowed Bi may form Bi_2_O_3_ which presents as a promoter for conductivity^25^. But Bi_2_O_3_ peak can not be found in Fig. 2A, and the trace amount may be responsible for the undetected peak. Meanwhile, this kind of substitution is not infinite because the extent of lattice distortion under perovskite structure is limited. Too much Na can enter into B-site, inducing a phase transition from cubic to rhombohedral. However, excessive Na cation probably serves as sintering aids, which promotes the effusion of Na from the lattice, causing the decrease of the lattice constant values since the ionic radius of Na is larger than that of Bi and Ti, which is in accordance with Table 1.

**Table 1:** Lattice constant and structure of Na_0.5+x_Bi_0.5_TiO_3−δ_ (x = −0.01–0.04).

Samples	Addr	x	Lattice Constant	Structure
Na_0.49_Bi_0.5_TiO_3−δ_	NBT-(-0.01)	−0.01	3.8763	Cubic
Na_0.50_Bi_0.5_TiO_3−δ_	NBT-0	0	3.8780	Cubic
Na_0.51_Bi_0.5_TiO_3−δ_	NBT-0.01	0.01	3.8846	Cubic - Rhombohedral
Na_0.52_Bi_0.5_TiO_3−δ_	NBT-0.02	0.02	3.8885	Rhombohedral
Na_0.53_Bi_0.5_TiO_3−δ_	NBT-0.03	0.03	3.8869	Rhombohedral
Na_0.54_Bi_0.5_TiO_3−δ_	NBT-0.04	0.04	3.8859	Rhombohedral

**Figure 3:**
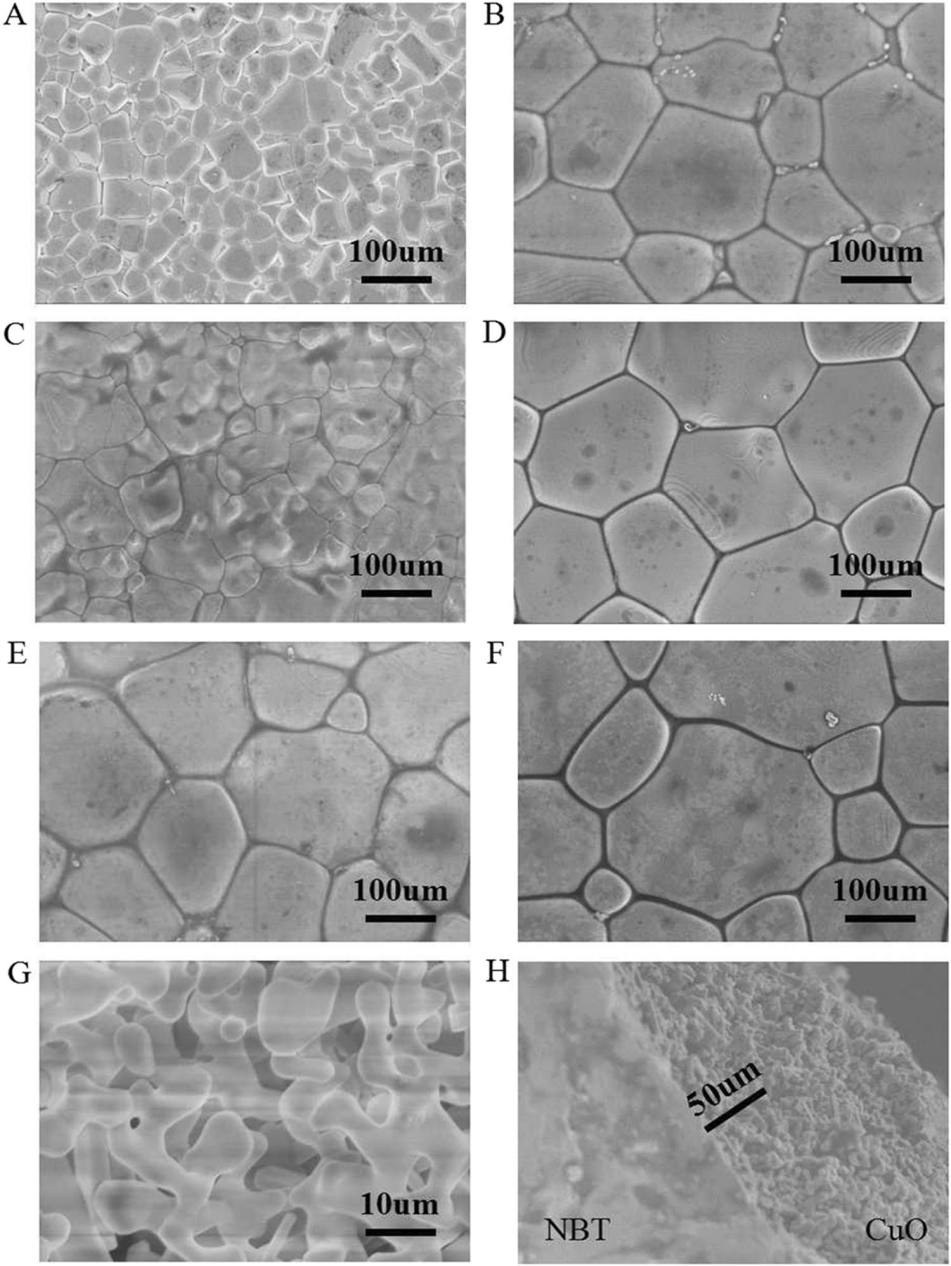
Scanning electron microscopic images of the surfaces of Na_0.5+x_Bi_0.5_TiO_3−δ_ substrates calcined at 1150 ^o^C for 2 h: (**A**) x = −0.01, (**B**) x = 0, (**C**) x = 0.01, (**D**) x = 0.02, (**E**) x = 0.03, (**F**) x = 0.04; (**G**) SEM images of the surface morphology of CuO sensing electrode, and (**H**) cross-sectional view of a NBT based sensor.

The surface morphologies of NBT electrolytes calcined at 1150 °C are depicted in Fig. 3A–F. It can be observed that NBT electrolyte is quite dense, and the grains with uniform sizes are all full-grown. The boundary between the NBT electrolyte and the CuO sensing electrode is clean. Cation nonstoichiometry may cause changes of grain size, for excessive Na cation probably serves as sintering aids, bringing down sintering temperature, thus altering the microstructure and the surface morphology, which is very good agreement with XRD results above^8^. With the ratio of Na cation varying from 0.49 (x = 0) to 0.54 (x = 0.54), the average grain size of Na_0.5+x_Bi_0.5_TiO_3−δ_ (x = −0.01, 0, 0.01, 0.02, 0.03, 0.04) increases from 135 µm to about 240 µm, indicating the excessive Na slightly promotes the grain growth, while the average grain size of Na_0.49_Bi_0.5_TiO_3−δ_ decreases to 40 µm, which demonstrates that the Na deficiency dramatically restrains the growth of grain. The surface photographs of CuO SE calcined at 800 °C for 3h and the cross-sectional view of the sensor are shown in Fig. 3G and H, respectively. It can be seen that the SE CuO is three-dimensional network due to the remove of graphite after calcined at 800 °C for 3 h. The 3D network CuO extends the length of the triple phase boundary (NO_2_/CuO/NBT) as well as promotes the adsorption of NO_2_ to CuO (SE), making the capture of electrons to NBT electrolyte easier, thus resulting in improved sensitivity of the sensor^26^.

**Figure 4:**
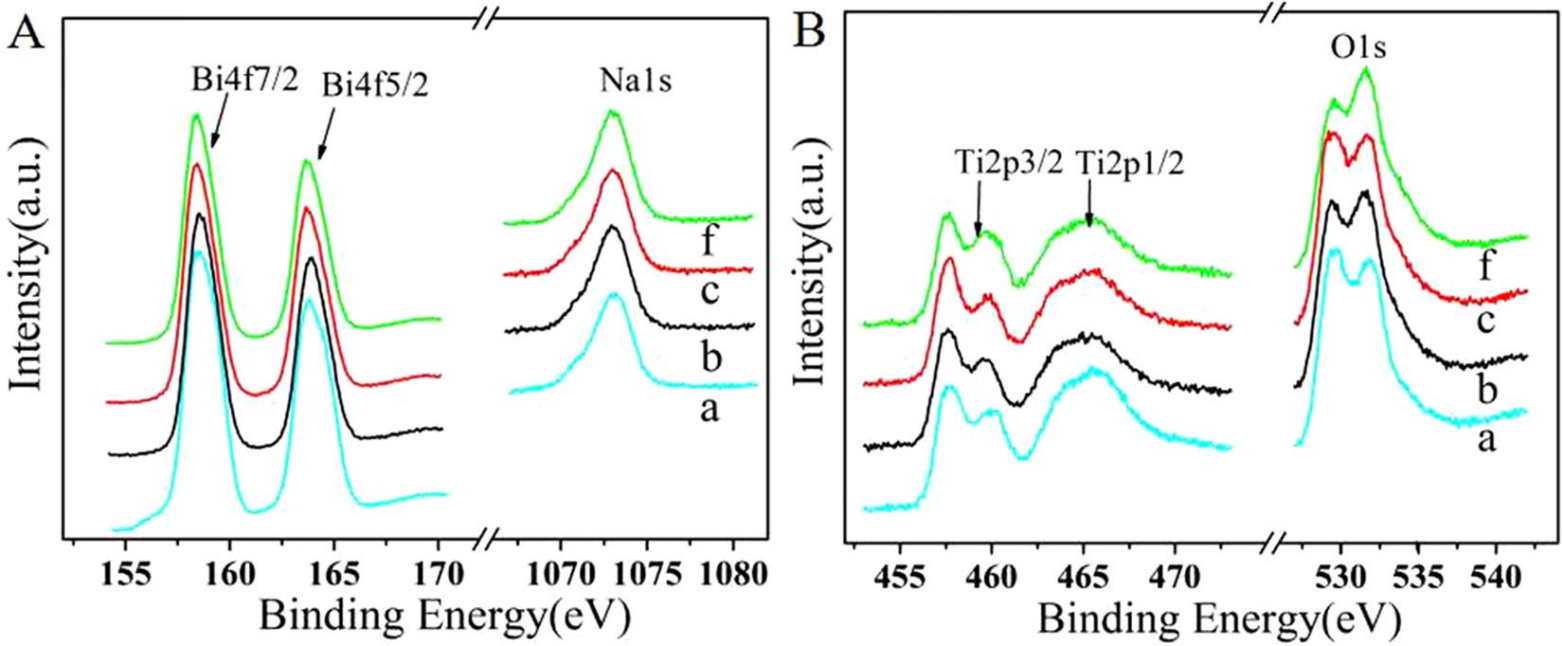
(**A**) X-ray Photoelectron Spectroscopy of Bi and Na of Na_0.5+x_Bi_0.5_TiO_3−δ_; (**B**) XPS of Ti and O of Na_0.5+x_Bi_0.5_TiO_3−δ_: (a) x = −0.01, (b) x = 0, (c) x = 0.01, (f) x = 0.04.

Fourier transform infrared spectroscopy and Raman spectra of Na_0.5+x_Bi_0.5_TiO_3−δ_ are depicted in Supplementary Fig. S1. XPS spectra of the Na_0.5+x_Bi_0.5_TiO_3−δ_ samples have been applied to investigate the surface chemical compositions and the states of the elements. According to Fig. 4A, the asymmetric component at around 157.3 eV and 162.4 can be identified as Bi4f spectrum, which corresponds to the Bi^3+^ other than Bi^5+^ of which appears at 158.8 or 164.2^27, 28^. An obvious Na1s peak at 1072 eV can be noticed, and there is no obvious peak at about 10.3 eV, suggesting that Na_2_O_2_ has not been formed^29^. It can be speculated that excessive Na enters into A-site and then overmuch Na into B-site of perovskite oxide, which can affect the valence of Ti, resulting in producing more oxygen vacancy. The XPS of Ti in Fig. 4B confirms the hypothesis. Two characteristic peaks of Ti2p1/2 (463.8 eV) and Ti2p3/2 (457.7 eV) are assigned to Ti^4+^ oxidation state^30^. However, as the increase of Na content, the Ti2p spectra are broadened and their intensities decrease slightly, while the splitting of Ti2p3/2 becomes distinct, indicating the formation of reduced oxidation states such as Ti^3+^ and Ti^2+^ species^30, 31^. The intensity ratio of Ti^3+^ or Ti^2+^ to Ti^4+^ of NBT-(−0.01), NBT-0, NBT-0.01, NBT-0.04 are different. The NBT-0.01 shows higher ratio of Ti^3+^ or Ti^2+^ to Ti^4+^ than that of others, which means NBT-0.01 possesses more Ti with reduced oxidation states, and this is a result of more oxygen vacancy. The O 1 s spectra could be fitted with three kinds of oxygen species. The oxygen peaks at 529.4, 531.6 and 533.8 eV are attributed to lattice oxygen, the electrophilic oxygen (e.g. O^2−^, O_2_^2−^, O^−^) and physically adsorbed or hydroxyl groups, respectively^32^. It can be seen that the change of Na content affects the ratio of lattice oxygen and electrophilic oxygen and the NBT-0.01 sample possesses more lattice oxygen which is related to oxygen vacancy.

**Figure 5:**
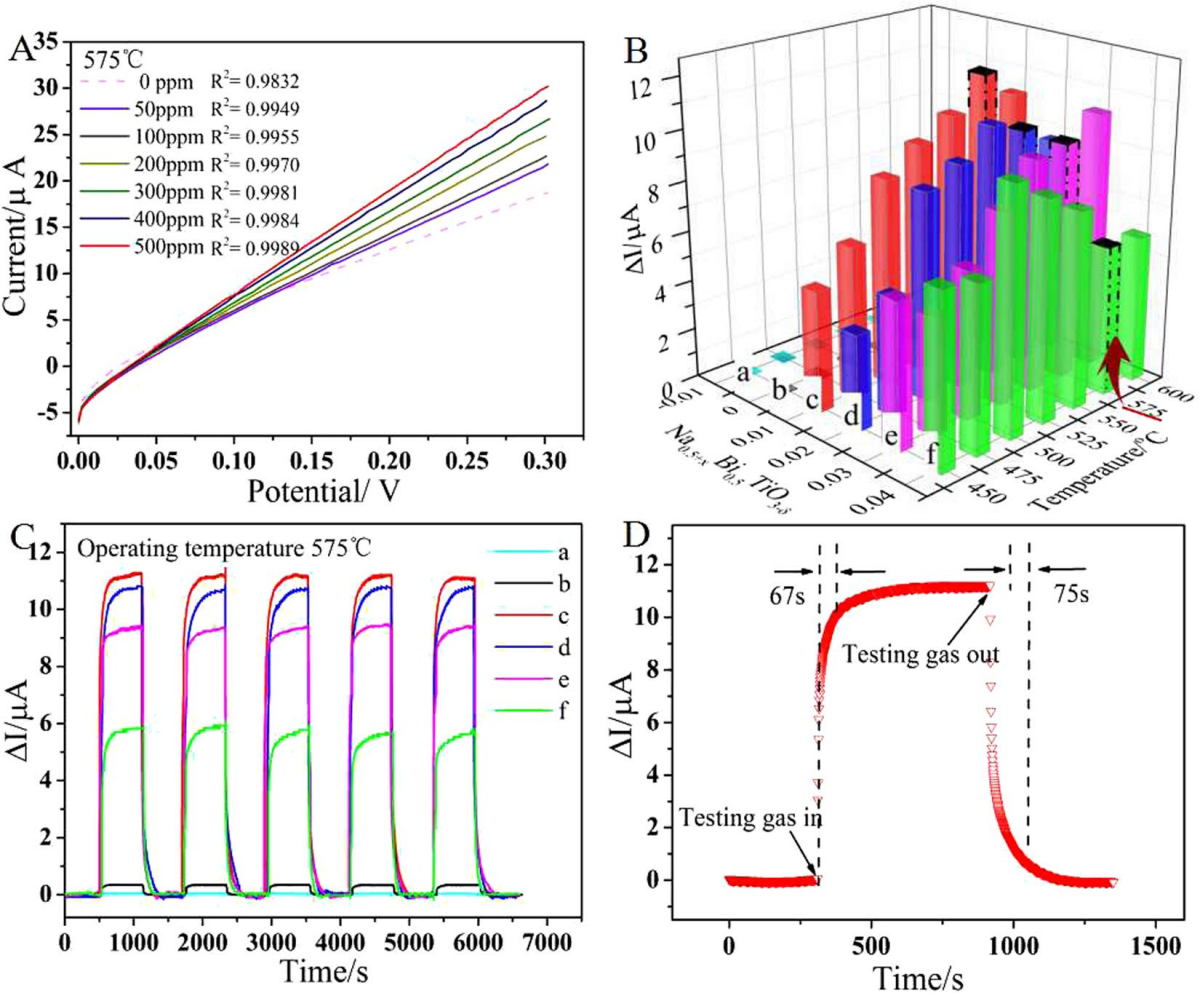
(**A**) The current-potential (*I-V*) polarization curves of the sensors based on Na_0.51_Bi_0.5_TiO_3−δ_ in 0–500 ppm NO_2_ at 575 ^o^C; (**B**) The response current values of the sensors based on Na_0.50+x_Bi_0.5_TiO_3−δ_ in 500 ppm NO_2_ at different temperature (450–600 °C); (**C**) Response transients of the Na_0.5+x_Bi_0.5_TiO_3−δ_ based sensors in 500 ppm NO_2_ at 575 °C in the presence of 5 vol% O_2_ (The applied potential is 300 mV, and the total flow rate is 400 mL/min): a~f refers to x = −0.01, 0, 0.01, 0.02, 0.03, 0.04, respectively; (**D**) Enlarged view of a typical procedure of Na_0.51_Bi_0.5_TiO_3−δ_ based sensor responses to the shift between testing gas (500 ppm NO_2_ + 5 vol. % O_2_ + N_2_ balance) and base gas (5 vol.% O_2_ + N_2_ balance) at 575 °C.

The representative current-potential (*I-V*) polarization curves in 0–500 ppm NO_2_ at 575 °C are displayed in Fig. 5A. The current values rise linearly as the voltage increases from 0 to 300 mV. The R^2^ values of fitting result of the *I-V* curves is close to 1, which testifies the linearity between the current values and the applied voltage in the range of 0–300 mV. This linear relationship between the current values and the applied voltage exists in all the testing temperatures and gas concentrations of the sensors in this work. Thus, 300 mV can be fixed as the constant polarization voltage in these Na_0.5+x_Bi_0.5_TiO_3−δ_ (x = −0.01,0,0.01, 0.02, 0.03, 0.04) series^33^.

The dependence of the response current values of all as-prepared sensors in 500 ppm NO_2_ at different operating temperatures on the various Na composition are depicted in Fig. 5B. The response current value is of vital importance for sensor because it reveals the detectability and accuracy of a sensor under real monitoring condition. Generally, the response current value ΔI = I_gas_ − I_base_ is regarded as the response signal of the amperometric-type sensor, where I_gas_ and I_base_ represent the current value of sensor in test gas and base gas, respectively. As seen from Fig. 5B, ΔI of NBT-(−0.01) based sensor is rather poor, whereas with Na content increase, NBT-0, NBT-0.01, NBT-0.02, NBT-0.03, NBT-0.04 based sensors show higher ΔI than that of NBT-(−0.01). Operating temperature can also influence the response value, sensitivity and stability etc, which are all key performance indicators to sensor. Here more comparison at 575 °C will be discussed. The response transients of the sensors based on NBT-x (x = −0.01, 0, 0.01, 0.02, 0.03, 0.04) electrolyte in Fig. 5C show well response-recovery characteristics to NO_2_ at 575 °C. Surprisingly, the sensor based on NBT-0.01 performs the highest ΔI values reaching up to 11.23 μA, which is about 354.26, 31.87, 1.05, 1.19, 1.89 times higher than those of the sensors based on NBT-(−0.01), NBT-0, NBT-0.02, NBT-0.03, NBT-0.04 at 575 °C, respectively. As well known, the O^2−^ generated on the TPB/sensing electrode would get through by electrolyte, so the more oxygen vacancies are, the higher the response current value ΔI is, which is in line with the characterization results of XPS. The lattice parameters and phase transition of XRD data also shows that the most obvious structural variation of NBT-x series happens on NBT–0.01. The slight Na that comes into A-site would bring about lattice distortion in A-site and Bi may be squeezed out as Bi_2_O_3_. The redundant Na enters into B-site, which makes the valence of Ti go down. The more Na enters into B-site, the more oxygen vacancies are produced. However, at the same time, overmuch Na in A-site might run out which makes Bi come back to A-site and the Bi_2_O_3_ disappear, as a result of the decrease of the conductivity and the response current value ΔI when Na content further increases. Therefore, the sensor based on NBT-0.01 with highest ΔI might be the optimal sensor compared to other ones. Response and recovery times are also important for sensors in practical application, which represent the real-time sensing efficiency. Figure 5D represents a typical period how the sensor based on NBT-0.01 responses to the shift between base gas (5 vol.% O_2_/N_2_ + N_2_ balance) and testing gas (500 ppm NO_2_/N_2_ + 5 vol.% O_2_/N_2_ + N_2_ balance). The current value increases dramatically soon after introducing the testing gas to the system and reaches a stabilized maximum value. After a short duration of time, base gas would be shifted back to the system, and then the current value decreases back to the original baseline value. Response time and recovery time are defined as the time a sensor needs to reach 90% of its maximum response value and decrease by 90% of its maximum response value, respectively^33^. It is visible that both the response time and recovery time of NBT-0.01 sensor are quite short, about 67 s and 75 s, respectively. The sensor is capable of a fair quick adsorption-response process of gas based on as prepared sensor, which could effectively shorten time of gas detection and shows important implications for practical application. The comparison of sensing performance of the sensor based on NBT-0.01, NBT-0 and YSZ with CuO SE is depicted in Supplementary Fig. S2, showing that NBT-0.01 based sensor exhibits better ΔI than that of YSZ under the same condition.

**Figure 6:**
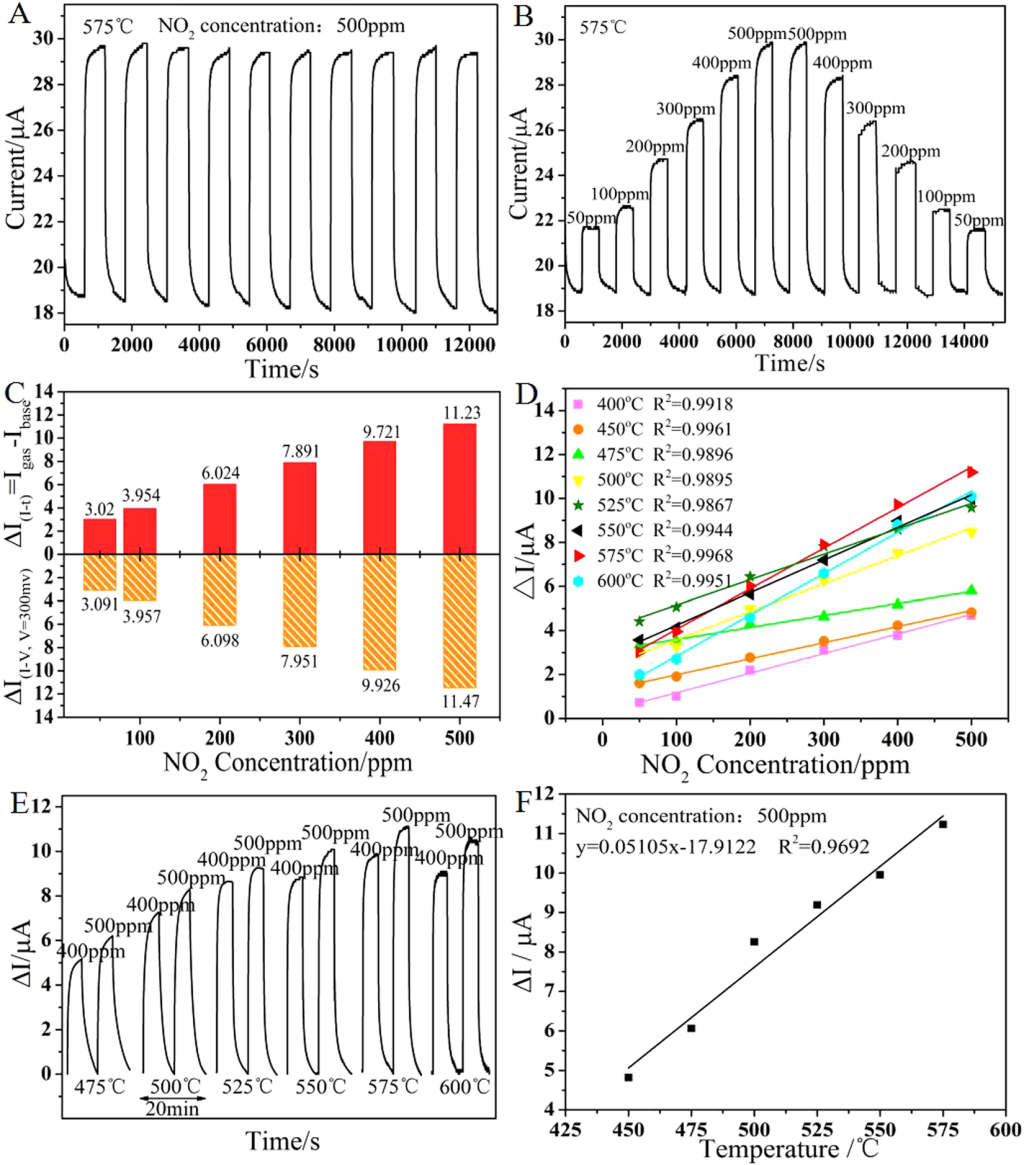
The response transients exposed to (**A**) 500 ppm NO_2_ and (**B**) various NO_2_ concentration at 575 ^o^C in the presence of 5 vol.% O_2_ (The applied potential is 300 mV, and the total flow rate is 400 mL/min); (**C**) The comparison between response current values calculated from I-t response transient curves (ΔI_(I-t)_ = I_gas_−I_base_) and the values subtracted from *I-V* polarization curves (ΔI′_(V=300mv)_ = I_(CONC,V=300 mv)_ − I_(0,V=300 mv)_) of the sensors based on Na_0.51_Bi_0.5_TiO_3−δ_ electrolyte; (**D**) The liner fit results of ΔI of the sensor based on Na_0.51_Bi_0.5_TiO_3−δ_ in a set of NO_2_ concentrations (50–500 ppm) at different temperatures (400–600 ^o^C); (**E**) The response transients to 400 ppm and 500 ppm NO_2_ at 475–600 ^o^C; (**F**) The liner fit result of ΔI of the sensor based on Na_0.51_Bi_0.5_TiO_3−δ_ in 500 ppm NO_2_ at 450–575 °C.

The response transients of the sensor based on NBT-0.01 to 500 ppm NO_2_ and 50–500 ppm NO_2_ at 575 °C are depicted in Fig. 6A and B. Obviously, the current value changes rapidly with the switching of NO_2_ concentration. Particularly, the mean squared error of ten times continuous responses to 500 ppm NO_2_ at 575 °C could be calculated to 0.052 μA, indicating the sensor can realize a stable and reproducible response towards NO_2_ gas^34^. The sensor based on NBT-0.01 would maintain its previous response value to the same NO_2_ concentration in Fig. 6B, without a clear variation during dozen of continuous detections to 50–500 ppm NO_2_ at 575 °C. It also could be observed that ΔI values of polarizing current values which corresponds to 300 mV in *I-V* curves (calculated from Fig. 5A) are approximately equal to ΔI values (ΔI = I_gas_ − I_base_) that were measured in *I-t* response transients profile on the certain NO_2_ concentration (calculated from Fig. 6B), and the comparison data are depicted in Fig. 6C. The comparison of the response current value in 50–500 ppm NO_2_ at different working temperatures between 400 and 600 °C are shown in Fig. 6D. The fitting result shows a superb linear relationship between the ΔI and NO_2_ concentrations at every temperature. The sensitivity of the sensor is defined as the slope of response current value ΔI on target gas concentration at a certain temperature, which can be calculated from the fitting results of ΔI on diverse NO_2_ concentration. And the sensitivities of the NBT-0.01 are 8.93, 7.31, 5.43, 12.71, 11.56, 14.83, 18.44, 18.35 nA/ppm at 400, 450, 475, 500, 525, 550, 575 and 600 °C, respectively. Basically, the sensitivity first increases and then decreases as the temperature elevating above 500 °C, but the sensitivities below 475 °C show a different trend. The reason of the sensitivities decrease with temperature elevating below 475 °C can be the low ΔI and the instability of catalytic effect of the sensing electrode. As operating temperature rising, catalytic effect of CuO enhances till excessively high temperature influences the gas reaction on the electrode. Obviously, the sensor based on NBT-0.01 obtains almost the highest sensitivity at around 575 °C.

Figure 6E exhibits the response transients of the NBT-0.01 based sensor towards 400 ppm and 500 ppm NO_2_ at 475 °C, 500 °C, 525 °C, 550 °C, 575 °C, 600 °C under a constant polarization voltage of 300 mV. It can be seen that the response signal to NO_2_ increases as temperature elevating and reaches the maximum at 575 °C, and then decreases with work temperature continuing to rise. The highest ΔI is 11.23 μA in 500 ppm NO_2_. When the temperature elevates, the catalytic activity of CuO SE enhances. However, excessive heat can accelerate the deactivation of electrode and the reaction between NO_2_ and other gas. Therefore, the optimal operating temperature of the NBT-0.01 based sensor to NO_2_ is about 575 °C. Moreover, the graph of Fig. 6F depicts the good liner fitting result of ΔI values and operating temperature in the range from 475 °C to 575 °C towards 500 ppm NO_2_. The great linear correlations are beneficial to practical gas sensing application.

**Figure 7:**
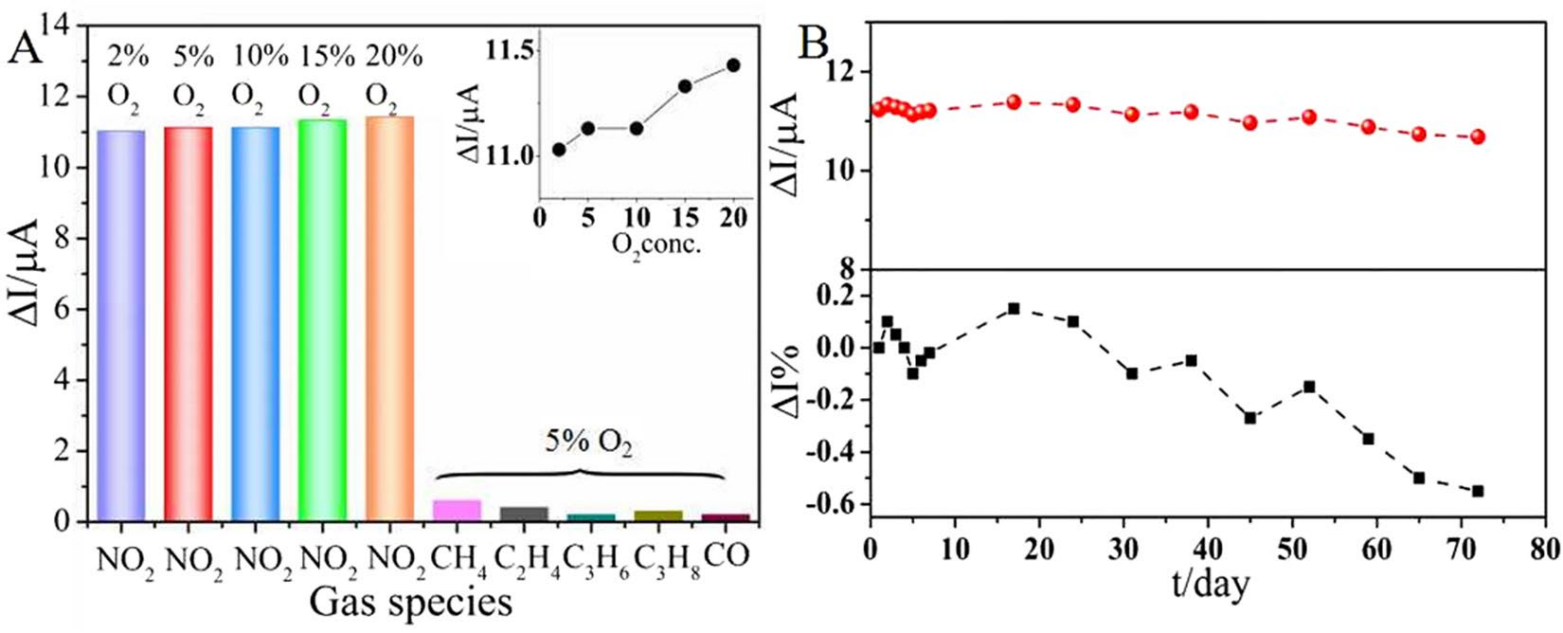
(**A**) Response current values of the sensor based on Na_0.51_Bi_0.5_TiO_3−δ_ towards 500 ppm NO_2_ with different O_2_ concentration (2–20%) at 575 °C, or 500 ppm CH_4_, C_2_H_4_, C_3_H_6_, C_3_H_8_, and CO in the presence of 5 vol.% O_2_ (The applied potential is 300 mV, and the total flow rate is 400 mL/min). The insert graph gives a more obvious variation trend of ΔI of Na_0.51_Bi_0.5_TiO_3−δ_ based sensor towards 500 ppm NO_2_ while O_2_ concentration changes; (**B**) The dependence of ΔI of the sensor based on Na_0.51_Bi_0.5_TiO_3−δ_ and the rate of changes on time in the presence of 500 ppm NO_2_ at 575 °C.

Actual pollutant released by industrials and motor vehicles might contain coexist waste gas, so it is necessary for us to evaluate the NO_2_ sensing performance in more variable conditions and compare the sensing performances with other coexist gas. A slight increase in the response current value is observed in Fig. 7A as O_2_ concentration increases from 2 to 20 vol.% of the mixed gas, revealing that the sensor has a good sensitivity to NO_2_ in the presence of 2–20 vol.% O_2_. The ΔI values of CH_4_, C_2_H_4_, C_3_H_6_, C_3_H_8_ and CO are 0.6, 0.4, 0.2, 0.3 and 0.2 μA, respectively. Compared with NO_2_ gas, ΔI values of these coexist gases could be ignored due to the extremely low ΔI values. It shows that the as-obtained Na_0.51_Bi_0.50_TiO_3−δ_ electrolyte based sensor possesses excellent selectivity towards NO_2_ at 575 °C. Meantime, good NO_2_ sensors should possess the ability to maintain a reliable stabilized sensing performance in a quite wide time period. The long term measurement was carried out during more than 2 months, with response tests carried out under a fixed interval. The variation trend of ΔI of the sensor to 500 ppm NO_2_ was displayed in Fig. 7B, and the corresponding experiment data of stability test could be found in Fig. S5. The response current value decreases by 0.55 μA, which only accounts for 4.9% of the original response current value 11.23 μA, exhibiting superior stability towards NO_2_ gas.

The interaction between the SE and target NO_2_ can be one of critical factors of the sensing performance. As well known, electron affinity of nitrogen dioxide is about five times higher than that of oxygen^35^, which would promote CuO SE to preferentially adsorb NO_2_ other than O_2_ or the other rest of gas among atmosphere. Adsorbed NO_2_ molecules could obtain electrons at the TPB of CuO electrode side, which accelerates the transport cycle of electron. The NO_2_-TPD plots for as prepared sensing electrode material CuO (see Supplementary Fig. S3A) verified that CuO can capture the NO_2_ molecule to its porous surface at about 260 °C, which would be in favor of the produce of O^2−^ at applied potential 300 mV, and quicken the dynamic response process of the sensor to NO_2_. The signal of off-gas of the NBT-0.01 sensor under 500 ppm NO_2_ at 575 °C is depicted in Supplementary Fig. S3B, which shows that NO and N_2_ might be resultant gases after the sensing behavior happened at the TPB, and the changes of other NO_x_ are too small to ignore, which is accord with the previous sensing mechanism.

## Conclusion

A novel highly sensitive NO_2_ sensor was fabricated using NBT electrolyte, CuO SE and Pt RE. The results indicated that the Na_0.51_Bi_0.50_TiO_3−δ_ based sensor exhibited the highest response current value (11.23 μA) in 500 ppm NO_2_ at 575 °C, which was higher than that of sensor based on YSZ electrolyte with CuO SE. The response current values were almost linear to NO_2_ concentrations in the range of 50–500 ppm at 400–600 °C with good response-recovery characteristics to NO_2_. The maximum sensitivity of Na_0.51_Bi_0.50_TiO_3−δ_ based sensor was 18.44 nA/ppm towards 500 ppm NO_2_ at 575 °C. The sensor presented excellent selectivity to other gases (CH_4_, C_2_H_4_, C_3_H_6_, C_3_H_8_, CO) and was barely not affected by coexistent O_2_ (2 to 20 vol.%) at 575 °C. The long-term stability was also relatively good with slight decrease of 4.9% of original ΔI values after 2 months. The performance of sensor based on non-rare-earth Na_0.51_Bi_0.50_TiO_3−δ_ electrolyte showed a potential application with low cost in motor vehicles or industrial processes.

## Methods

### Preparation of NBT Electrolytes and CuO Sensing Electrode

The citrate sol-gel method was applied to synthesize Na_0.5+x_Bi_0.5_TiO_3−δ_ (x = −0.01, 0, 0.01, 0.02, 0.03, 0.04) powders. Analytical grade NaNO_3_, Bi(NO_3_)_3_·5H_2_O, tetrabutyl titanate, Cu(NO_3_)_2_·3H_2_O and citric acid were used as raw materials. Tetrabutyl titanate was first blended with absolute alcohol with full stir. NaNO_3_ and Bi(NO_3_)_3_·5H_2_O was dissolved in water and 10–15% dilute nitric acid aqueous solution, respectively. Then the Bi(NO_3_)_3_·5H_2_O nitric acid solution was slowly added into tetrabutyl titanate ethyl alcohol mixture, achieving a stable mixed solution. NaNO_3_ aqueous solution was mixed with above mentioned solution, obtaining the metal salt mixed solution that contains Na, Bi and Ti cation. After that, a certain amount of citric acid was dissolved into deionized water in a beaker, in which the molar ratio of citric acid to metal cation is equal to 1.25: 1. An appreciate amount of aqueous ammonia was dripped into the citric acid solution to adjust the pH value to 7–9. At last the metal salt mixed solution was added into the citric acid-ammonia solution with a slow speed under an on-going stir at 80 °C for 1 h to generate a transparent, yellowish precursor solution. Meanwhile the continued heating and rabbling at 80 °C were carried on for another 10 h to form a wet sol. The wet gel was dehydrated in an oven at 150 °C for 24 h to form a black dry gel. The dry gel was pulverized and then heated in an oven at 270 °C for 10 h. The powder was calcinated at 650–700 °C for 2 h in air followed by a second calcined at 850–900 °C for 2 h, and the calcined Na_0.5+x_Bi_0.5_TiO_3−δ_TiO_3−δ_ powder was light-yellow. Then the calcined powder was pressed into discs of 8 mm in diameter and 2 mm in thickness with a uniaxial steel die followed by an isostatically press procedure at 200 Mpa. Finally, the compacted discs were sintered at 1150 °C for 2 h in air, and the Na_0.5+x_Bi_0.5_TiO_3−δ_ electrolyte substrate was obtained.

The synthesis of the sensing electrode used the same citrate sol-gel method. First, Cu(NO_3_)_2_·3H_2_O was added into ethyl alcohol and stirred at 40 °C for 1 h, then citric acid was added into the above mixture with continuous rabbling at 60 ^o^C for 5 h to generate a thick sol. After that the sol was dehydrated in an oven at 150 ^o^C for 10h to form a bluish green dry gel. At last, the gel was pulverized and calcined at 500 ^o^C for 3 h in air.

### Characterization and Fabrication of the NBT sensor

The XRD data were collected by X-ray powder diffractometer (XRD, PANalytical, *X*’Pert PRO) in the range of Bragg angle 2θ (10°–100°) with Co Kα radiation (λ = 1.78901 Å) at a scan rate of 10° min^−1^ with a step size of 0.0167° at room temperature, then revised by Cu Kα (λ = 1.5405 Å). The microstruture images of Na_0.5+x_Bi_0.5_TiO_3−δ_ electrolyte were characterized by field emission scanning electron microscopy (SEM, Hitachi, S-4800). The infrared spectra in the range of 1200–400 cm^−1^ were recorded on Fourier-transform infrared spectroscopy device (FT-IR, Thermo Fisher Scientific, Nicolet 6700). The samples were prepared as KBr pellets. The Raman spectra were recorded in backscattering geometry using the 532 nm line of an Ar-ion laser covering the range 40–1000 cm^−1^ (Raman, Renishawn, in Via Reflex) in room temperature and fitted by Lorentzian area function using the JANDEL Peakfit software. A 20× objective was used to focus the laser beam on the sample. X-ray Photoelectron Spectroscopy (XPS) was performed on a Thermo Scientific ESCALAB 250 spectrometer with a monochromatic Al-K_α_ source (K_α_ = 1486.6 eV). The data were calibrated by the binding energy of C1s (284.6 eV) as the standard. NO_2_ Temperature-programmed desorption (NO_2_-TPD) was carried out on an AutoChem 2920 instrument equipped with a thermal conductivity detector (TCD). The signal was monitored by a mass spectrometer. Before the measurement, 0.05 g of the as prepared CuO sample powder was pretreated with 2000 ppm NO_2_/He (30 mL/min) at 300 ^o^C for 30 min, and then cooled down to room temperature. After the sample was treated by a He purge (30 mL/min) for 30 min, the CuO sample was heated from 40 to 700 ^o^C.

An amperometric-type NO_2_ sensor based on NBT electrolyte with CuO SE and a Pt RE is fabricated and depicted in Fig. 1. The CuO SE and Pt RE paste were coated on the two sides of the NBT-x electrolyte by Screen-printing technology and fired at 800 ^o^C. Then the Pt wires (0.2 mm) were cohered on the both side of disc and sintered at 800 ^o^C.

### Sensing performance evaluation

The apparatus used for sensing properties evaluation is displayed in Supplementary Fig. S4, which is made of a gas flow adjustment and readout assembly, a heating quartz tube furnace with programmable temperature controller that capable of setting temperature from 0 to 800 ^o^C, and an electrochemical workstation (Zahner, IM6) connected to a computer to record electrochemical signal. The total flow of the gas atmosphere was set to 400 ml/min, composed of an alterable concentration of NO_2_ (0–500 ppm), O_2_ (0–20 vol%) and two-way balance gas N_2_ to maintain a constant total flow without inordinateness. The flow rates of the gases were controlled by mass flow meters. As the gas flow was switched from base gas (5 vol.% O_2_/ N_2_ + N_2_ balance) to testing gas (NO_2_, CH_4_, C_2_H_4_, C_3_H_6_, C_3_H_8_ or CO + 5 vol.% O_2_/ N_2_ + N_2_ balance), the sensing properties change can be dynamically recorded by electrochemical workstation. The current-potential (*I-V*) curves of sensors were measured by potential dynamic method at a scan-rate of 10 mV/s in the voltage range from 0 to 300 mV using a two-electrode configuration. The response transient curves of the sensors were measured by potentiostatic method at a fixed 300 mV.

The trace signal of off-gas of sensor was performed on a mass spectrometry (Dycor Dymaxion, DME200MS). The sensor was placed in testing tube with Pt wires connected to IM6 electrochemical workstation. The applied potential is 300 mV, and the total flow rate is 400 mL/min. The testing gas (500 ppm NO_2_/He + 5 vol.% O_2_/ He + He balance) and base gas (5 vol.% O_2_/ He + He balance) were used to avoid interfering by N_2_ in normal mixed gas.

## Electronic supplementary material

Supplementary Information
