# Supplementary material for: A Novel Highly Sensitive NO2 Sensor Based on Perovskite Na0.5+xBi0.5TiO3−δ Electrolyte
Source: Sci Rep. 2017 Jul 10;7:4997. doi: 10.1038/s41598-017-05169-4 (PMC5504058; doi:10.1038/s41598-017-05169-4)
Supplement: Supplementary file 1 — Supplementary Information [file 41598_2017_5169_MOESM1_ESM.pdf]

Supplementary Information

A Novel Highly Sensitive NO_2 Sensor Based on Perovskite $Na_{0.5+x}Bi_{0.5}TiO_{3-\delta}$ Electrolyte

Yihong Xiao[a], Chufan Zhang[a], Xu Zhang[a], Guohui Cai[a],Yong Zheng[a], Ying Zheng[b], Fulan Zhong*[a], Lilong Jiang*[a]

[a]National Engineering Research Center of Chemical Fertilizer Catalyst (NERC-CFC), School of Chemical Engineering, Fuzhou University, Gongye Road No.523, Fuzhou 350002, Fujian, P. R. China

[b]College of Chemistry and Materials Science, Fujian Normal University, Fuzhou, China

Email: zhongfulan@fzu.edu.cn; jll@fzu.edu.cn

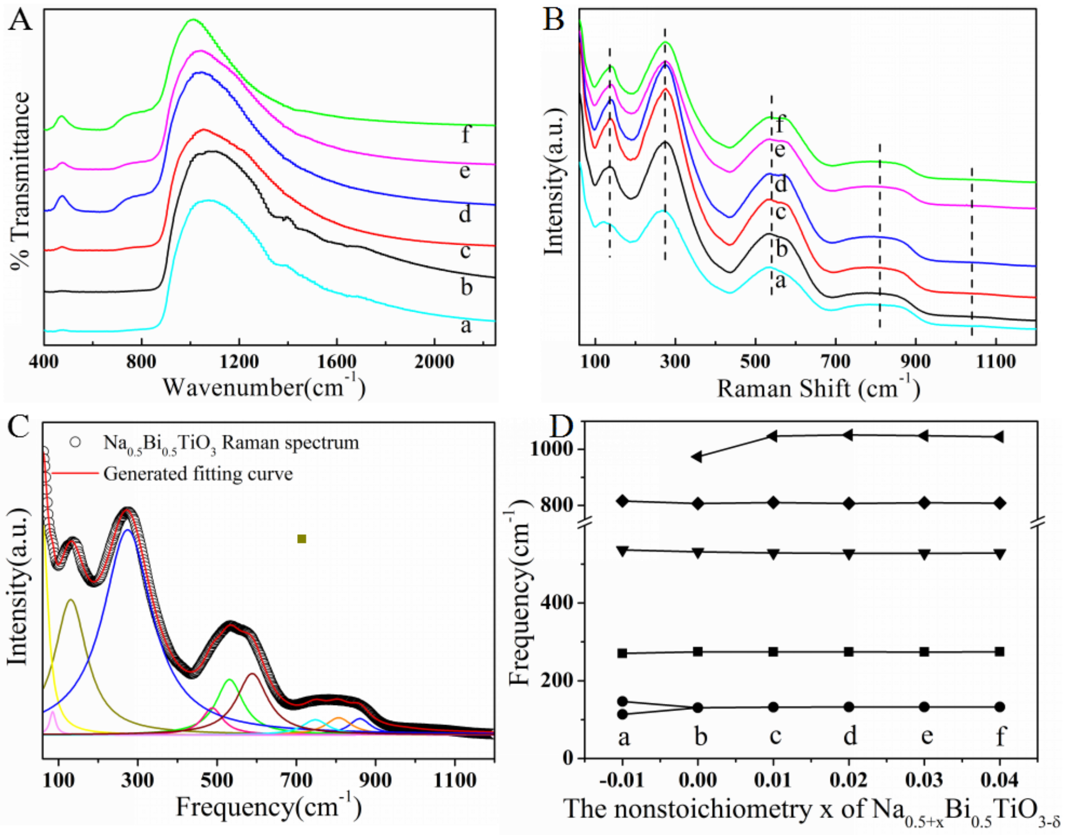

Figure S1. (A) Fourier transform infrared spectroscopy of $Na_{0.5+x}Bi_{0.5}TiO_{3-\delta}$; (B) Raman spectrum of $Na_{0.5+x}Bi_{0.5}TiO_{3-\delta}$; (C) Results fitted by Lorentzian area function of $Na_{0.5}Bi_{0.5}TiO_3$ Raman spectrum; (D) Change of typical fitting peak position for different nonstoichiometry on $Na_{0.5+x}Bi_{0.5}TiO_{3-\delta}$: (a) x = -0.01, (b) x = 0, (c) x = 0.01, (d) x = 0.02, (e) x = 0.03, (f) x=0.04.

Figure S1A shows Fourier transform infrared spectroscopy of $Na_{0.5+x}Bi_{0.5}TiO_{3-\delta}$ powders calcined at 1150 °C, and the typical oxygen metal bonds exist in the range of 600-800 cm^{-1}. The bands around 600 cm^{-1} and 810 cm^{-1} could be attributed to the characteristic vibration of Ti-O octahedron and the stretching vibration of Bi-O, respectively[1,2]. The absorption peaks of Ti-O and Bi-O bonds become stronger and shift toward the lower wave number with the increment of Na cation ratio, indicating that the change of Na cation ratio has induced the transformation within crystalline structure but the $A_{1/2}A_{1/2}BO_3$ perovskite phase maintains its structure[3].

The Raman spectra in Figure S1B have been systematically decomposed into individual Lorentzian components, which are located at 59.78, 85.08, 130.67, 274.37, 487.99, 531.56, 588.65,

747.98, 806.78, 860.37 and 973.38 cm^{-1} (Figure S1C). The (TO_3) mode situated in the region of 532 cm^{-1} can be deconvolved into three Raman peaks which are associated with the TiO_6 octahedral vibration, and it is a typical mode in metal oxides of typical-perovskite structure[4]. The A_1 (TO_1) mode at about 131 cm^{-1} is related to the Na-O vibration or distorted octahedral [BiO_6] and [NaO_6] clusters. The (LO_3) mode near 807 cm^{-1} involves the site within the rhombohedral lattice which contains octahedral distorted [TiO_6] clusters. And the mode near 1000 cm^{-1} has been reported to relate to oxygen vacancy4[-6]. Taking a close look to the peak positions and intensities of these modes, transformation could be found in Figure S1D. The strength of peak at about 131cm^{-1} and 274.37cm^{-1} are observed to depress with x decreases, and a split of peak can be found near 131 cm^{-1} with x= -0.01. As the Na content increases, the mode shifts toward the lower wave number. The changes can be explained as the variation of Na cation ratio induces disorder in A-site3. Furthermore, we can notice that the mode at about 1000 cm^{-1} disappears at x = -0.01 and the peak position varies with x elevating, indicating the oxygen vacancy of NBT-(-0.01) is relative low. The peak position shifts first toward higher wave number till x=0.01, and then shifts toward lower wave number as Na content increases from x=0.02 to x=0.04. This means that the change of Na content causes the difference of oxygen vacancy and the NBT-0.01 sample exhibits quicker speed in conducting oxygen ion.

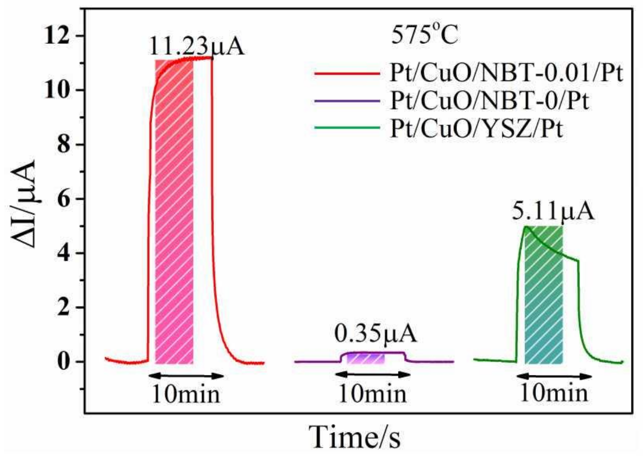

Figure S2. The response transients of sensors based on NBT-0.01, NBT-0 and YSZ electrolyte with CuO sensing electrode towards 500 ppm NO_2 at 575°C (The applied potential is 300 mV. The total flow rate is 400 mL/min, and the percentage of oxygen is 5 vol.%).

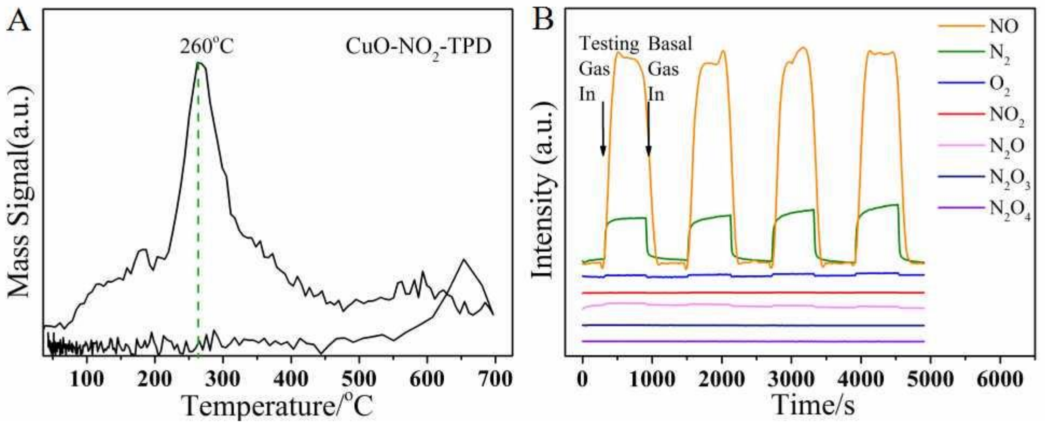

Figure S3. (A) NO_2-TPD signals of as prepared CuO. The CuO powder was first pretreated with 2000ppm NO_2/He (30 mL/min) at 300 °C for 30min and cooled down to room temperature. Then the CuO sample was treated by a He purge (30 mL/min) for 30 min, followed by a heat process from 40 to 700 °C; (B) Mass spectrometer signals corresponding to different gases (NO, N_2, O_2, NO_2, N_2O, N_2O_3, N_2O_4) of off-gas of test quartz tube with the sensor based on $Na_{0.51}Bi_{0.5}TiO_{3-\delta}$ placed in it. The $Na_{0.51}Bi_{0.5}TiO_{3-\delta}$ based sensor is exposed to testing gas (500 ppm NO_2/He + 5 vol.% O_2 + He balance) and base gas (5 vol.% O_2 + He balance) at 575 °C (The applied potential is 300 mV, and the total flow rate is 400 mL/min).

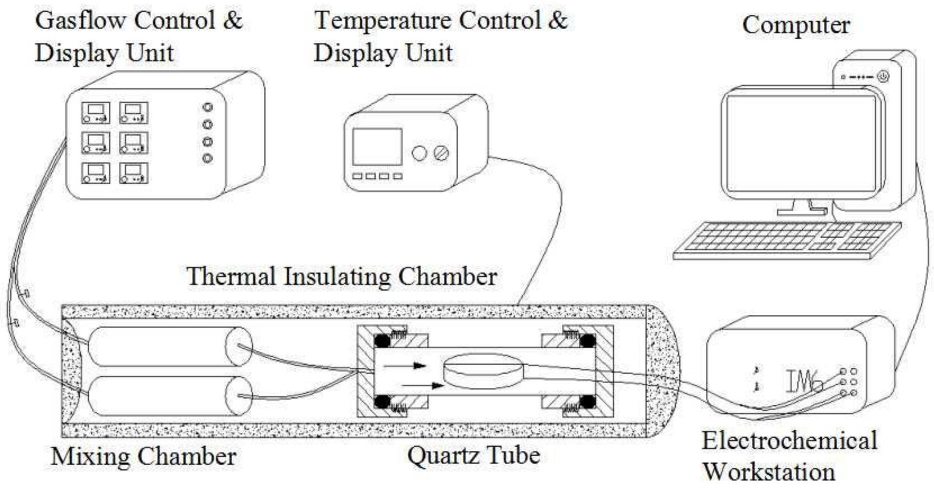

Figure S4. The device of sensing performance evaluation.

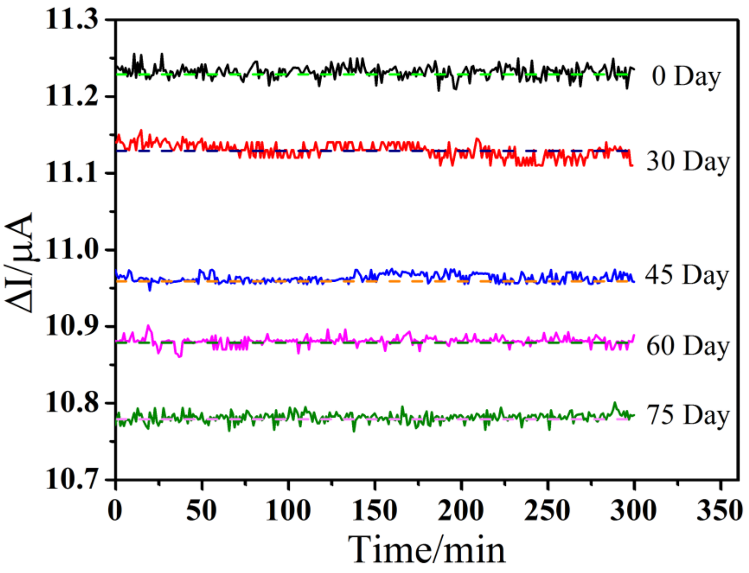

Figure S5. Stability test for the sensor based on $Na_{0.51}Bi_{0.5}TiO_{3-\delta}$ substrate at 575 ℃ in the presence of 500 ppm NO_2 (The applied potential is 300 mV, and the total flow rate is 400 mL/min).

1. Wang, Y. & Santiagoavilés, J. J. Synthesis of lead zirconate titanate nanofibres and the Fourier-transform infrared characterization of their metallo-organic decomposition process. *Nanotechnology* **15**, 32-36 (2004).

2. Liao, M. *et al*. Nd-substituted bismuth titanate ferroelectric nanofibers by electrospinning. *J. Cryst. Growth* **304**, 69-72 (2007).

3. Yoko, T., Kamiya, K. & Tanaka, K. Preparation of multiple oxide $BaTiO_3$, fibres by the sol-gel method. *J. Mater. Sci.* **25**, 3922-3929 (1990).

4. Parija, B., Badapanda, T. & Panigrahi, S. Morphotropic Phase boundary in BNT-BZT solid solution: A study by Raman spectroscopy and electromechanical parameters. *J. Ceram. Process Res.* **16**, 565-571 (2015).

5. Barick, B. K., Mishra, K. K., Arora, A. K., Choudhary, R. N. P. & Pradhan, Dillip. K. Impedance and Raman spectroscopic studies of $(Na_{0.5}Bi_{0.5})TiO_3$. *J Phys. D Appl. Phys.* **44**, 355402-355411 (2011).

6. Tripathy, S. N., Mishra, K. K., Sen S. & Pradhan, D. K. Dielectric and Raman Spectroscopic Studies of $Na_{0.5}Bi_{0.5}TiO_3$–$BaSnO_3$ Ferroelectric System. *J. Am. Ceram. Soc.* **97**, 1846–1854 (2014).
